# How Natural Language Processing Can Aid With Pulmonary Oncology Tumor Node Metastasis Staging From Free-Text Radiology Reports: Algorithm Development and Validation

**DOI:** 10.2196/38125

**Published:** 2023-03-22

**Authors:** Sander Puts, Martijn Nobel, Catharina Zegers, Iñigo Bermejo, Simon Robben, Andre Dekker

**Affiliations:** 1 GROW School for Oncology and Reproduction Maastricht University Medical Centre+ Maastricht Netherlands; 2 Department of Radiation Oncology Maastro Maastricht Netherlands; 3 School of Health Professions Education Maastricht University Maastricht Netherlands; 4 Department of Radiology and Nuclear Medicine Maastricht University Medical Center+ Maastricht Netherlands

**Keywords:** radiology, reporting, natural language processing, free text, classification system, oncology, pulmonary, clinical decision, clinical

## Abstract

**Background:**

Natural language processing (NLP) is thought to be a promising solution to extract and store concepts from free text in a structured manner for data mining purposes. This is also true for radiology reports, which still consist mostly of free text. Accurate and complete reports are very important for clinical decision support, for instance, in oncological staging. As such, NLP can be a tool to structure the content of the radiology report, thereby increasing the report’s value.

**Objective:**

This study describes the implementation and validation of an N-stage classifier for pulmonary oncology. It is based on free-text radiological chest computed tomography reports according to the tumor, node, and metastasis (TNM) classification, which has been added to the already existing T-stage classifier to create a combined TN-stage classifier.

**Methods:**

SpaCy, PyContextNLP, and regular expressions were used for proper information extraction, after additional rules were set to accurately extract N-stage.

**Results:**

The overall TN-stage classifier accuracy scores were 0.84 and 0.85, respectively, for the training (N=95) and validation (N=97) sets. This is comparable to the outcomes of the T-stage classifier (0.87-0.92).

**Conclusions:**

This study shows that NLP has potential in classifying pulmonary oncology from free-text radiological reports according to the TNM classification system as both the T- and N-stages can be extracted with high accuracy.

## Introduction

### Background

Staging patients with cancer is of utmost importance to determine the most appropriate treatment regime to ensure the best outcome for the patient. The tumor, node, and metastasis (TNM) is an internationally accepted clinical classification system and a standard for the proper staging of patients with cancer [1]. Radiological imaging by means of a chest computed tomography (CT) scan is an important pillar for the TNM classification in clinical practice. Because the radiological report is the way to communicate observations to referring clinicians, the content of the report needs to be complete and accurate [2-4]. Specifically, the tumor (T), the node (N), and the metastasis (M) status should be known. However, the radiological report is in most cases still a free-text report, in which layout, structure, readability, and accuracy largely depend on the reporter.

### Prior Work

Natural language processing (NLP) can be applied to extract specific information from free text. This can also be applied to radiological reports when, for instance, specific coding and structured reporting are not used [5,6]. Already several studies have been performed using NLP in radiology, and implementation in clinical practice seems just a matter of time [7-9]. Existing NLP implementations in radiology are often related to extracting data for cancer registries, such as oncological follow-up, tumor recurrence rates, and follow-up of critical oncological findings [10-13]. In addition, nononcological studies have been performed using NLP to search for specific statements from pulmonary angiography reports, and imaging reports of subdural hematoma in the acute setting or, more generally, to extract recommendations from radiology reports [14-16].

NLP has also been used in a recent and ongoing transnational project to extract the stage in pulmonary oncology from free-text radiological chest CT scan reports [17,18]. The overall goal is to build a language-independent algorithm that can extract pulmonary oncology staging according to the TNM classification.

In prior work, a rule-based NLP algorithm was trained and validated on Dutch radiological reports before it was translated and validated on English reports, which showed an accuracy rate for T-stage ranging between 0.84 and 0.87 [18] The rule-based approach is thought to be the easiest way to accurately determine the oncological stage, as TNM is already a rule-based system. When, for instance, only machine learning (ML) strategies for staging were used, apart from the issue of correctly finding the specific concepts, the algorithm also needs to extract the set of rules of each concept from the training data, which requires a very large amount of data.

### Goal of This Study

This paper describes the process of training and validation of extraction of the N-stage of pulmonary oncology of Dutch free-text radiological chest CT reports and discusses whether this is a feasible tool in addition to the already validated rule-based T-stage algorithm.

### Hypothesis

For adequate staging, the N-stage should also be known. We hypothesize that, as the items to build the N-stage should be mentioned in the same radiological staging report as used to classify the T-stage, it should be possible to accurately extract the N-stage from the report using a similar process as previously used for the T-stage.

## Methods

### Corpus Description

For this study, radiological reports of diagnostic chest CT scans used for the staging of pulmonary oncology were used. The training and validation sets consisted of, respectively, 95 and 97 reports to provide sufficient variety for training and detail for validation.

Reports were included when a primary pulmonary malignancy was described by a radiologist. The included free-text radiological reports have been constructed by several different radiologists, other than the authors, using a speech recognition tool (G2 Speech). Exclusion criteria were (1) restaging and follow-up reports, (2) cases with 2 primary tumors, and (3) incomplete reports. The included reports were independently classified by 2 authors (MN and SP) according to the eighth TNM classification system [1]. For every report, the T-stage and N-stage were labeled. Because TNM-stage was not specified in the radiological report, this had to be done manually. Annotation guidelines were set for proper and consistent labeling, see annotation guidelines in Multimedia Appendix 1. Tumor stage characteristics of both groups are shown in cohort composition of the training and validation set (Table 1). The layout of the included reports differed and contained one or more of the following subheadings: clinical details, description of the modality, report, body part, and impression.

The training set was used to identify the content of the radiological report to find the appropriate synonyms used for reporting N-stage. These synonyms were used to build new N-staging rules that were incorporated in the existing T-stage rule-based algorithm.

**Table 1:** Class label distribution on the training and validation set. Reports were independently classified by 2 authors (MN and SP) according to the eighth TNM^a^ classification system.

TNM-stage	Training (N=95), n	Validation (N=97), n
T1aN0	0	0
T1aN1	0	0
T1aN2	1	0
T1aN3	0	0
T1bN0	7	2
T1bN1	2	0
T1bN2	1	1
T1bN3	0	1
T1cN0	7	9
T1cN1	0	3
T1cN2	2	5
T1cN3	3	1
T2N0	0	1
T2N1	0	0
T2N2	4	3
T2N3	1	1
T2aN0	4	5
T2aN1	2	2
T2aN2	2	3
T2aN3	3	1
T2bN0	4	2
T2bN1	0	2
T2bN2	4	6
T2bN3	3	2
T3N0	5	5
T3N1	1	0
T3N2	6	9
T3N3	4	6
T4N0	7	8
T4N1	0	2
T4N2	13	11
T4N3	9	6

^a^TNM: tumor, node, and metastasis.

### Ethical Considerations

This research was submitted to the Medical Ethical Board of the Maastricht University Medical Center. They confirmed that this research is not subjected to the Medical Research Involving Human Subjects Act (non-WMO). Therefore, approving this research and waiving informed consent. Only retrospective and anonymized data were used.

### Determining T-Stage

**Figure 1:**
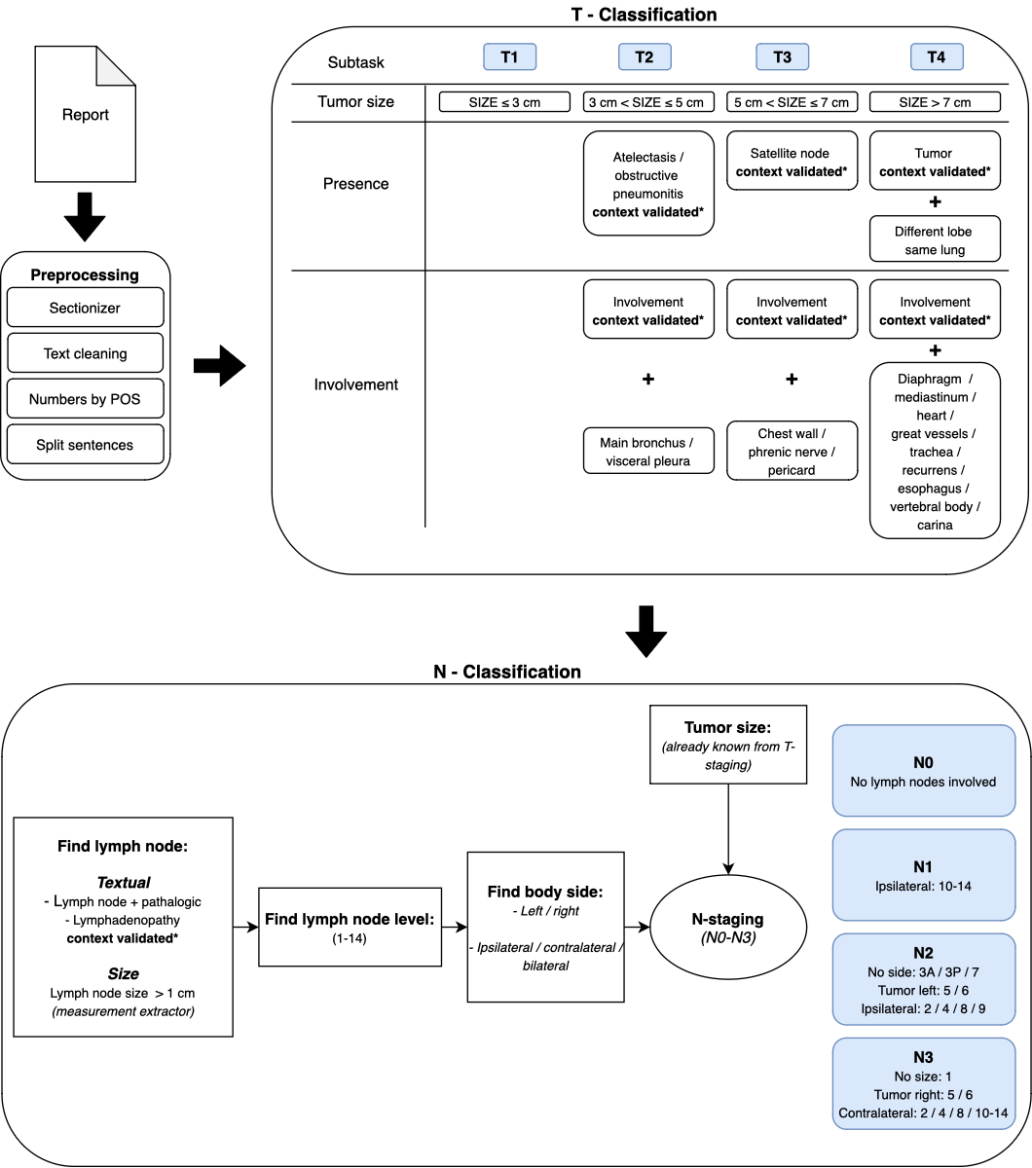
Schematic overview of the rule-based T- and N-staging algorithm. In the preprocessing step, raw data of the report are prepared for actual processing. The processing is divided into T-stage and N-stage in which several subtasks are displayed to finally stage the pulmonary tumor and pulmonary lymph nodes. N: node; POS: part-of-speech; T: tumor.

In this study, the same rule-based TNM-stage algorithm was used as published earlier, with preprocessing steps, such as sectionizing, text cleaning, extracting numbers, and accurate sentence splitting [17]. The processing steps were based on the extraction of 3 items that are important for T-staging using regular expression (RegEx): size, presence, and involvement (Figure 1). After extracting all relevant findings items for tumor staging, a separate rule-based classifier is used for final classification. Outcomes were used for T-staging the tumor (Multimedia Appendix 2).

### Determining N-Stage

For extracting the N-stage of pulmonary oncological cases, the eighth TNM classification was analyzed in detail, and 4 items were recognized as important: (pathological) lymph node, lymph node level, lymph node side, and tumor side, see the schematic overview of the rule-based T- and N-staging algorithm in Figure 1. To accurately stage the N-stage, the described lymph nodes had to be found and matched with their potential context first to know whether or not the lymph node was a pathological lymph node. Therefore, synonyms of N-specific concepts, such as “lymph nodes” and “pathological,” had to be found to build a specific RegEx per concept. Therefore, lymph node–specific rules had to be built.

For N-staging, it was necessary to look more extensively at the relation between “context target” and the “context modifiers” because it appeared that a pathological lymph node was less specifically mentioned in the report than the primary tumor. A target could, for instance, be the concept for the word “lymph node” and the modifier the adjective, stating it is “enlarged.” Additionally, an enlarged lymph node could be described not only by text but can also be highlighted by quantifying its enlarged size. This resulted in finding three distinct ways of mentioning the pathological lymph node in which, (1) “lymph node” and “pathological lymph node” had to be extracted, (2) “lymph node” and its pathological size had to be extracted, and (3) “lymph node” and the word “pathological” had to be matched, and a specific RegEx had to be built per item. Regular expressions related to the context that indicates the presence or absence of target concepts (context validation), such as negations and uncertainty, could be reused from the T-staging process, but the additional category for “pathological” had to be added.

Subsequently, the lymph node level had to be found and, since there are 14 different thoracic levels, a RegEx was built per level. Furthermore, the side of the tumor and the pathological lymph node had to be extracted to define the lymphadenopathy to be ipsilateral, contralateral, or bilateral. This was not necessary for extraction of the T-stage, and specific rules for sentence analysis had to be set. The size of the lymph node was extracted by the measurement extractor component, which uses the number category of the open-source part-of-speech tagger as input. The measurement extractor extracts and normalizes measurements from text, which is, for example, required for expressions such as 12×44×29 mm. Finally, the tumor side was matched to the side of the pathological or enlarged lymph node and used for definitive N-staging, see concept synonyms in Multimedia Appendix 2.

### Statistical Analysis

For both the training and the validation sets, the substage accuracy scores were calculated separately for the T-stage and the N-stage. T-substage is a subdivision of the T-stage to provide more detail, for example, stage T1 (≤3 cm) contains substage T1c (2 to ≤3 cm) [1,18]. Next to the T- and N-stage, the combined accuracy score (TN-stage) was scored for the training and validation sets. To find out whether the N-stage extension in this TN-classifier compromised the T-stage outcomes, the earlier version of the algorithm, which was a T-stage classifier only, was also run on the training and validation sets. In addition, the accuracy score was calculated when only tumor size was taken into account.

Confusion matrices were created for the training and the validation sets to visualize the performance of the T-stage, N-stage, and TN-stage classification. In addition, the precision (ie, specificity), recall (ie, sensitivity), and *F*_1_-score (ie, combined metric for precision and recall) for the combined TN-stage classifier were calculated for the training and validation sets. Weighted scores were used, metrics were first calculated by label, and then averaged using the addition of weights corresponding to the number of true instances for each label. Different types of errors were grouped by category for further analysis: data selection, context extraction, concept extraction, and reporter errors. In category data selection, errors are grouped related to extracting relevant sections and sentences, as executed by the sectionizer. Concept errors are related to extracting concepts; entities are linked to ontologies (Multimedia Appendix 2). Concept errors are split into missing synonyms, ambiguation, and complexity errors. Failing to extract implicit information is an example of a complexity error. Context errors are divided into missing synonyms and matching errors, a matching error occurs when the context is linked to the wrong concept. Reporter errors are related to user-or-speech input.

### Graphical User Interface

**Figure 2:**
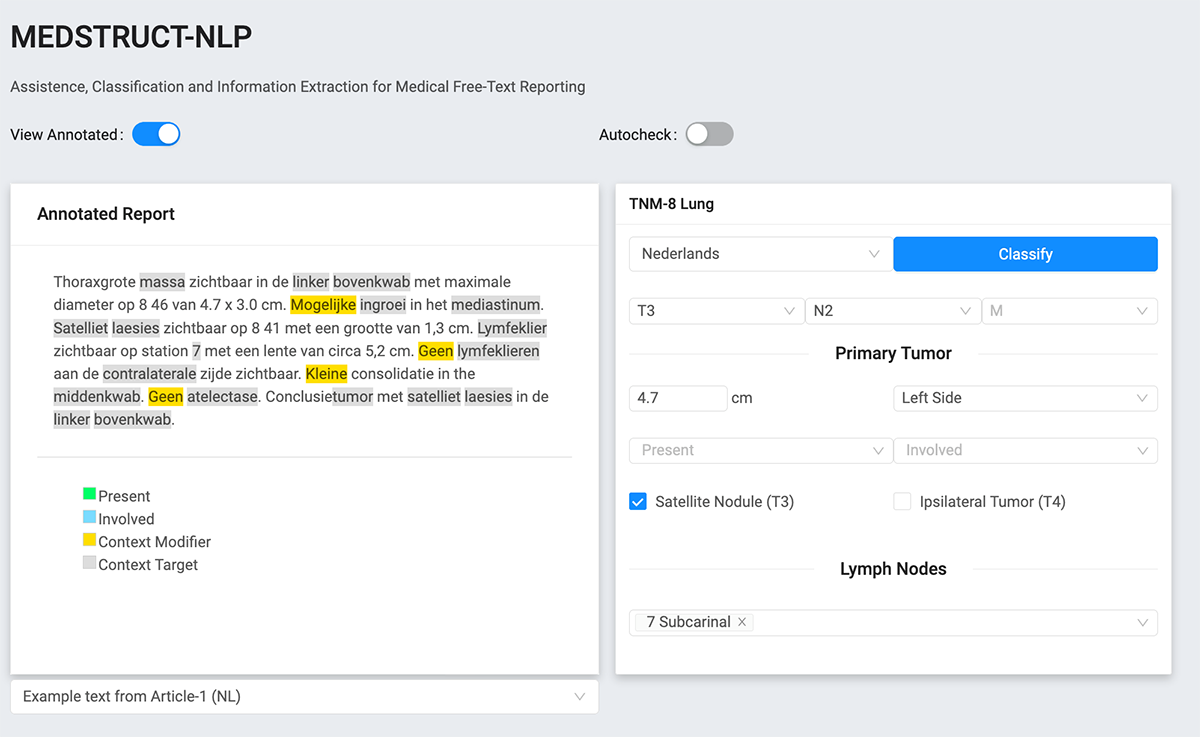
Graphical user interface MedStruct. M: metastasis; N: node; T: tumor; TNM: tumor, node, and metastasis.

A graphical user interface (GUI) was extended to highlight the TN-stage of the report in the staging screen, see graphical user interface MedStruct in Figure 2. When the N-rules were set, they have been implemented in this tool to help with the staging check by visualizing the scored TN-stage by the algorithm and comparing those with the manually extracted TN-stage.

## Results

**Figure 3:**
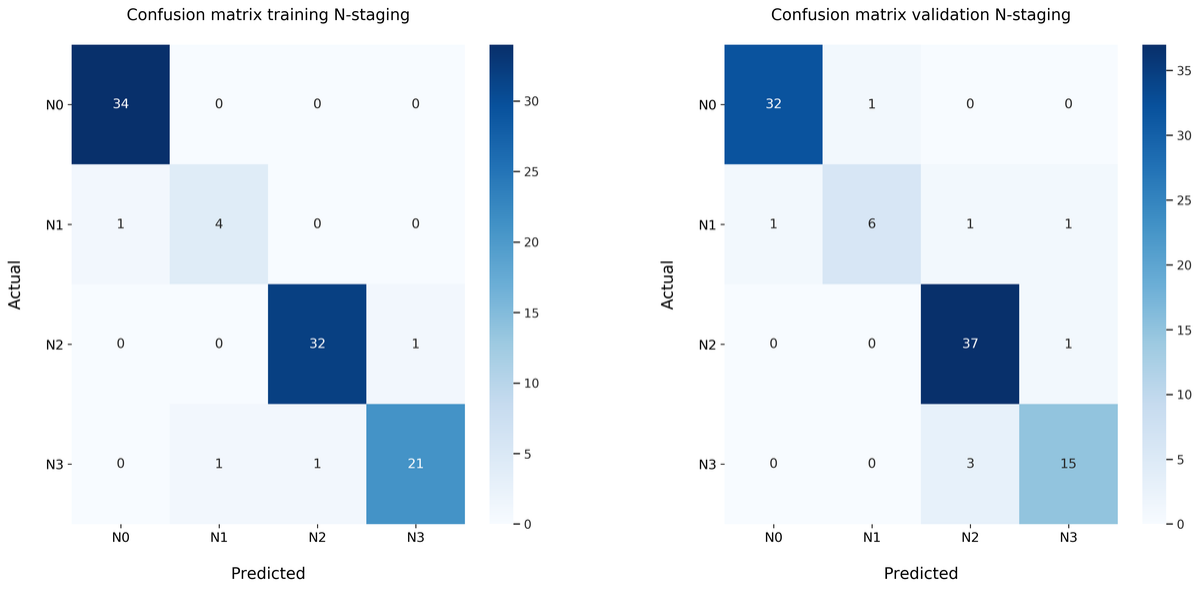
Confusion matrices of the N-stage classification only on the (left) training set and (right) validation set. N: node.

**Figure 4:**
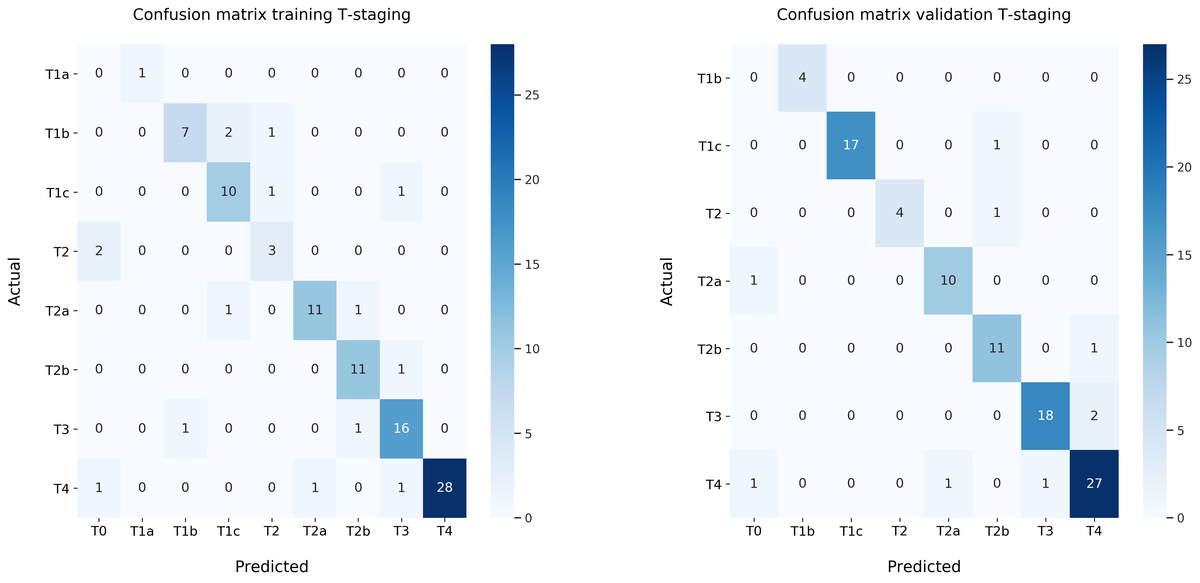
Confusion matrices of the T-stage classification only on the (left) training set and (right) validation set. T: tumor.

**Figure 5:**
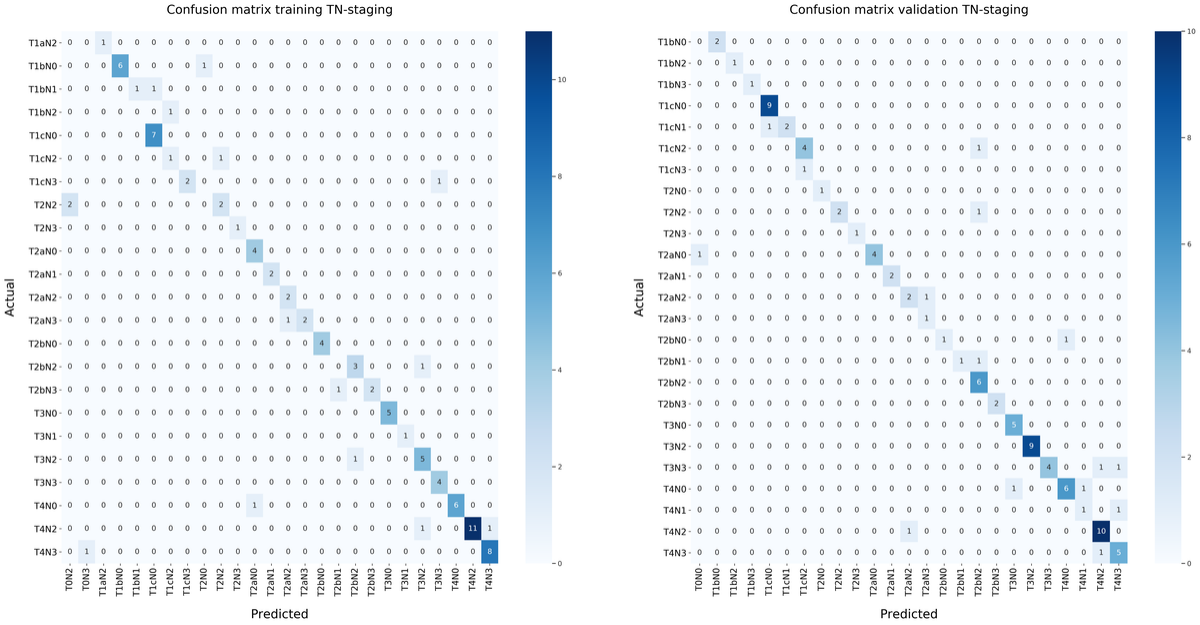
Confusion matrices of the TN-stage classification on the (left) training set and (right) validation set. N: node; T: tumor; TN: tumor and node.

The accuracy rates for the T-stage score were 0.84 and 0.92, respectively, for the training (N=95) and validation (N=97) set. N-stage accuracy scores were 0.96 and 0.92, respectively, for the training and validation sets. The combined accuracy TN-stage scores were 0.84 and 0.85, respectively, for the training and validation sets, see T-, N-, and TN-stage classifier accuracy in Table 2. Confusion matrices are created for the N-stage (Figure 3), T-stage (Figure 4), and the combined TN-stage (Figure 5) on both training and validation sets. Looking at the combined outcomes of the training and validation confusion matrices, it can be observed that the N-stage outcome was understaged in 7 cases and overstaged in 5 cases. T-stage outcome was understaged in 9 cases and overstaged in 11 cases out of the grand total of 192 cases. The TN-stage confusion matrices show that in both sets 15 cases were wrongly classified, and that in total, 14 cases were understaged and 16 overstaged. In addition, the errors made were equally divided between both sets. The weighted precision, recall, and *F*_1_-score for the combined TN-stage classifier are shown in Table 3. The errors found were categorized into specific subcategories as shown in TN-stage errors by category (Table 4). In total, 16 errors were made in the training set and 16 in the validation set leading to 15 classification errors, with 1 error in both T- and N-stage and 1 case in both the training and the validation set.

**Table 2:** T-,^a^ N-,^b^ and TN^c^-stage classifier accuracy. Accuracy scores of the training and validation sets of the separate T-stage and N-stage and the combined TN-stage. For comparison, the T-classifier outcomes are shown for the current sets as well as the T-stage for only tumor size.

TNM^d^-subclassification	TN-classifier		T-classifier	
	Training (N*=*95)	Validation (N*=*97)	Training (N*=*95)	Validation (N*=*97)
Accuracy T-stage (T-substage)	0.87	0.92	N/A^e^	N/A
Accuracy N-stage	0.96	0.92	N/A	N/A
Accuracy TN-stage	0.84	0.85	N/A	N/A
Accuracy T-stage (size only)	0.80	0.81	0.76	0.79
Accuracy T-stage (T-stage)	0.89	0.93	0.82	0.86

^a^T: tumor.

^b^N: node.

^c^TN: tumor and node.

^d^TNM: tumor, node, and metastasis.

^e^N/A: not applicable.

**Table 3:** Weighted precision, recall, and F1-scores of the TN^a^-stage.

Partition	Precision	Recall	*F*_1_-score
Training (overall)	0.89	0.84	0.86
Validation (overall)	0.87	0.85	0.84

^a^TN: tumor and node.

**Table 4:** TN^a^-stage errors by category.

Error group and error type and description			Training (N=95), n		Validation (N=97), n
**Data selection—Sectionizer**					
	Subheadings not present or falsely not found—falsely correlation tumor or nodal description	2 (1T,^b^ 1N^c^)		2 (2T)	
**Context extraction—Missing**					
	Context not matched because of missing modifier in configuration	1 (1T)		3 (2T, 1N)	
	Stated uncertainty not found accurately	1 (1T)		3 (2T, 1N)	
**Context extraction—Complexity**					
	Context mismatch, wrong modifier detected	4 (3T, 1N)		0	
	Abdominal para-aortal lymph node	0		1 (1N)	
**Concept extraction—Missing**					
	Pathological synonym	0		1 (1N)	
**Concept extraction—Ambiguity**					
	Nodal description or station	2 (1T, 1N)		0	
	Tumor-dependent atelectasis	2 (2T)		0	
	Pulmonary vein	1 (1T)		0	
**Concept extraction—Complexity**					
	Size description	1 (1T)		1 (1T)	
	T4 multiple lobes—implicit mentioning	1 (1T)		1 (1T)	
	Side implicit mention	0		1 (1N)	
	Mention nodal status	1 (1N)		0	
**Reporter—Wrong input**					
	Typing or speech error	1 (1T)		2 (1T, 1N)	
	Incomplete node mentioning (location or pathological)	0		3 (3N)	
	Inconsistent tumor location	0		1 (1T)	
	Total errors	16^d^		16^d^	

^a^TN: tumor and node.

^b^T: tumor.

^c^N: node.

^d^16 errors in total, leading to 15 wrong classification scores.

## Discussion

### Principal Results

The aim of this research was to build an NLP algorithm to classify pulmonary oncology as reported in free-text radiological CT chest staging reports according to the eighth TNM classification. In addition to previously developed T-staging rules, specific N-staging rules were added to the algorithm in order to find 4 additional items necessary for proper N-staging: (pathological) lymph node, lymph node level, lymph node side, and tumor side.

The accuracy scores for the N-stage were 0.96 and 0.92, respectively, in the training and validation set. This shows that this rule-based approach and the rule’s set are viable for extraction of the items necessary for proper N-staging. From the combined TN-stage accuracy scores for the training and validation set, 0.84 and 0.85, respectively, it can be observed that outcomes are a bit lower. However, taking into account that both the T-stage and the N-stage had to be correct, accuracy is still reasonably high and comparable with outcomes of the T-stage alone (0.87-0.92, see Table 2). The outcomes of the accuracy score of the TN-classifier are comparable or slightly better than the accuracy score of the T-classifier only, showing that the addition of the N-stage rules did not interfere with the overall outcome.

When looking at the error categories, a total of 20 errors were made in the T-stage classification and 12 in the N-stage classification. One case in both groups, the N-stage and the T-stage, was falsely staged. Overall, many different errors occurred, which shows the heterogeneity of the reports, and hence, the extent of the task to tackle and optimize this rule-based approach. The T-stage classifier is validated in another language at an external institute [18]. From the external validation and the current N-stage validation, we can conclude that the rules do not seem to under or overfit, and the performance on all training and validation sets is similar.

The difference between the high N-stage accuracy scores and the low TN-stage accuracy scores is slightly compromised by difficulties still experienced by the T-staging rather than by N-staging difficulties (Table 4). This can be explained by the fact that the T-staging process is more difficult to accurately perform with a rule-based approach and may therefore be less reliable than the N-staging. This is not surprising when looking at the number of substages used in the T-stage compared to the N-stage, with 8 substages for the T-staging (T1a-T4) versus 4 substages for the N-staging (N0-N3). In addition, the T-staging rules include several exceptions and are therefore more extensive than the N-staging rules [19]. This is illustrated by the fact that only the location of the pathological lymph node is different in the N-staging process, whereas for the T-staging, tumor size, presence, and involvement differ per stage. Furthermore, to accurately T-stage the tumor, the size is of utmost importance leading to accuracy scores of 0.80 and 0.81 when only finding the accurate tumor size. The additional scores of 0.07 and 0.11 are achieved by setting multiple rules, which is a laborious process.

### Rules Versus ML

Although rule-based algorithms are in general inflexible and difficult to generalize, a rule-based approach has been chosen over an ML approach. First, the classification problem is multiclass (8 T-stages and 4 N-stages), which requires a large amount of data to cover all classes. Where an ML approach learns from examples, a rule-based approach does not require examples for each class, as knowledge from other sources such as a knowledge expert (eg, radiologist) can be implemented. Next to the number of classes, numerous combinations of findings can result into a single class. The N-stage, for example, depends on the combination of tumor laterality and lymph node location. Additionally, the free-text reports contain medical jargon, context, and writing styles, all contributing to the variety of the data. Learning such a complex classification task by an ML approach requires, and therefore a large, annotated data set, which is unfortunately difficult to obtain. TNM-stages registered in the electronic health record are determined by a multidisciplinary team. The staging outcome of the multidisciplinary team can differ from staging exclusively based on imaging; therefore, all reports need to be manually labeled. Retrieving reports from the electronic health record meeting inclusion criteria and labeling radiology reports with TNM-stage require expert knowledge and are very time-consuming. As the rule-based algorithm is mainly “learned” from knowledge experts and only partly from data, an equal data split ratio is selected. For both sets, approximately 100 reports were annotated to provide variety for training and detail for validation.

Second, as the official TNM-staging system is a rule-based classification system, rules are known by forehand, and the implementation of the algorithm can almost be a one-to-one translation of the official rules. Learning rules, such as size thresholds for T-stage classification, is counterintuitive if the thresholds are already predefined. Adapting the rule-based algorithm between TNM versions is straightforward, as differences between versions are often minor alterations of the rules [20]. A rule-based approach can be easily adapted to those differences, while a data-dependent ML approach would require relabeling a large amount of data.

Third, explainability is often required for usage in a clinical setting. To explain the classification, the outcome of each TNM-rule feature should be explained. For example, N-staging depends on the laterality of the tumor; to determine the N-stage, tumor laterality should be provided.

When an ML approach would be used for explainability, it would likely be better to divide the classification tasks into subtasks (no end-to-end classification). The outcome of the subtasks could be used as an input for a rule-based system. Several ML approaches extract named entities from radiology reports [21,22]. Named entity recognition could be seen as a subtask for staging, although errors made by our approach in extracting concepts (entities) are limited (see Table 4).

Finally, external validation has shown that the rule-based T-staging algorithm is generic enough to be easily translated to another institute and language [18]. Language and other content differences can be managed by editing the configuration and without altering the implemented rules.

### Future Research Suggestions

To increase the overall TN-outcome, both T-staging and N-staging processes should be improved. However, it is thought that the accuracy of the T-staging is limited, even with finding more synonyms using this single rule-based approach. At this point, changing the rules of the classification process is a tradeoff between improving one rule while decreasing the outcome of the other. Instead, it may be better to improve the T-staging outcomes with ML by, for instance, specific training to find difficult-to-extract concepts or match the right context. For example, accuracy may then be improved better by identifying gravity-dependent atelectasis, matching uncertainty mentions to the correct concepts, or finding specific T4 exceptions.

In a single radiological report, often several (pathological) lymph node stations are described. This is beneficial for an NLP algorithm, since, even when a pathological lymph node is missed, another pathological lymph node (in the same level or leading to the same stage) may be picked up by the algorithm, thereby not changing the final outcome. Additionally, the word “lymphadenopathy” is highly specific for pathological lymph nodes, and so are its modifiers (location, ipsilateral, contralateral, or bilateral). Such “backups” and specific terms are less present in primary tumor staging.

Fortunately, the combination of concepts for “pathological contralateral lymph node” or “enlarged supraclavicular lymph node” is quite specific for the N3-stage, which, in turn, allows for better extraction. When a pathological lymph node in this specific location is found, other lymph node stations are of less importance as the highest N-stage is reached. This can be the explanation for the high combined accuracy score of 97.2% in the N2-stage, in which N2 harbors the most lymph node levels. The same may be true for the high combined N0-stage accuracy score of 98.5%, in which the accurate distinction is to properly match negations, and the rules set to not match any of the pathological concepts, enlarged sizes, or the word “lymphadenopathy” to the lymph node levels. Although this “backup” may be beneficial for the final results, the algorithm still needs to highlight all pathological lymph nodes correctly to increase the accuracy of the radiological report.

However, the abovementioned is not true when only 1 pathological lymph node is present in a random (nonspecific) location. Perhaps, this more specific task or option is the reason that relatively many errors are present in the N1- and N3-staging groups with overall 0.71 and 0.87 accuracies compared to 0.99 and 0.97 for the N0- and N2-stages. In addition, for N3 nodes, the contralateral side needs to be accurately distinguished. Another challenging problem occurs when several lymph nodes are described within a single sentence, which complicates the correct matching of contextual information (eg, uncertainty and negation) even more.

To increase specific N-stage accuracy scores, dependency relations could be used to have a better idea of which contextual property belongs to which lymph nodes; in case multiple lymph nodes are present in a single sentence, this may improve the accuracy score further. In addition, also ML-based NLP implementation might be helpful to find specific terms and mentions. This seems less difficult to train than the T-staging because the rules are less difficult. This implementation of ML should be targeted at full matching between the lymph node and all its mentioned properties such as pathological state, level, and nodal size.

The implementation of TN-algorithm should be externally validated in a different institute as well as in different languages as done for the T-stage algorithm [18]. The system is currently deployed in the authors’ institute for evaluation purposes on a voluntary basis, a prospective study could result in more insights on the clinical value of the approach.

### Standardization

An approach to increase accuracy, without artificial intelligence tooling and without IT interference, can be through standardization of the report. This standardization step, which is specifically not a template or a structured report, represents a set of simple rules on how to report. This can be as simple as stating the size of the tumor or the pathological lymph node directly after the stated concept, perhaps between brackets. A different option is to only give sizes for pathological lymph nodes and tumors, or mention only 1 lymph node level per sentence. Alternatively, the primary tumor or specific lymph node with all its highlights is reported in 1 sentence. This way, the set of rules will result in higher accuracy scores. In addition, the readability of the reports will improve as well even without difficult and extensive interventions during the reporting process. The GUI that has been developed allows real-time analysis, and feedback to the reporter may also be beneficial here. As mentioned earlier, this overall quality report–enhancing step can also be achieved by ML only but requires a vast number of reports to train all variants. Also, more annotated data are then needed and, as this is laborious for training purposes, hence not desirable.

### Human Input Errors

When looking at the total errors in the training and validation sets, the reporter error group “reporter” is responsible for 21.9% of the total errors. These errors are caused by incomplete or inaccurate information, or speech or typing errors when, for instance, adjusting the report. It is difficult for a rule-based algorithm to find these errors because staging information can be implicated in the text rather than explicit mentioning, and typographical errors or speech errors occur in many different ways. Even with manually determining classification from the reports, it was sometimes difficult to interpret the correct classification. Knowledge of the reporting and staging process, for instance, order-specific information, overall contextual information, or knowing what item to prioritize in wrongly stated concepts or context, made it possible to determine the correct stage. These errors cannot be solved with a rule-based approach, and it is questionable whether ML will do better, as these errors may not occur systematically—even in a vast number of reports—hampering the ability of ML to recognize these.

### Real-Time Feedback

It is very interesting to see whether reporter-induced errors can be diminished when the report is staged live, and outcomes are displayed using a GUI, as these errors are relatively easy to prevent. From the grand total of 192 scans, 7 staging errors could have been prevented. It seems that an improvement in reporting skills, combined with the implementation of specific ML, would increase accuracy outcome scores the most.

### Benefits of the Approach

Doctors can, if they prefer, use free text over structured reporting, while structured data can be stored. Free text allows nuances and the possibility to provide additional information, which would not be captured by structured reporting. NLP combined with live feedback to the end user could result in additional quality assurance (QA) steps. The QA step could stimulate the use of a standardized vocabulary, which makes reports more consistent and comprehensible. A QA step could enforce the end user to provide certain required information. Finally, the structured output of the algorithm could be used (live) as an input for other algorithms; for example, algorithms that provide suggestions or decision support.

### Limitations

One of the limitations of this study is that there were only 192 cases included. The cohort composition of the training and validation set (Table 1) shows that only a subset of the 32 TN-stages was present in the training and validation set. The relatively few reports included in these groups induced heterogeneity. Another limitation is that the outcomes of the additional N-stage were only based on reports from 1 institution. Future work requires its external validation. In addition, positron emission tomography (PET)-CT reports were not included in this study, in which possibly important tracer uptake information is missed. This is mainly important to exclude enlarged lymph nodes without uptake and include small lymph nodes with tracer uptake.

To complete fully assisted TNM-staging, M-stage is also needed. This is, however, expected to be much more difficult or may be even not feasible at all. For instance, brain metastasis can only be seen with high accuracy on brain magnetic resonance imaging. In addition, whole body PET-CT is used as a screening tool to search for distant metastasis. However, suggested distant metastasis on PET-CT mostly requires additional, specifically targeted imaging to confirm metastasis. As such, only metastasis located in the chest can be found on a staging chest CT, and only those can be staged. In future research, PET-CT reports need to be validated. Merging information from different radiological staging reports is needed for accurate full TNM-staging. Perhaps, such an algorithm can be useful for and applied to oncology staging forms or multidisciplinary meetings.

### Conclusions

NLP shows its potential in classifying pulmonary oncology from free-text radiological reports according to the TNM classification system, as both the T- and N-stages can be extracted with high accuracy. Integration with ML approaches to perform specific tasks should improve accuracy scores even more. However, standardization of the reporting manner and a visual check by the reporter before finalizing the report may be relatively easy implementations in clinical practice to increase accuracy.

## Appendix

Multimedia Appendix 1 Annotation guidelines.

Multimedia Appendix 2 Concept synonyms.

## Abbreviations

CT: computed tomography
GUI: graphical user interface
ML: machine learning
NLP: natural language processing
PET: positron emission tomography
QA: quality assurance
RegEx: regular expression
TNM: tumor, node, and metastasis
