# Supplementary material for: How Natural Language Processing Can Aid With Pulmonary Oncology Tumor Node Metastasis Staging From Free-Text Radiology Reports: Algorithm Development and Validation
Source: JMIR Form Res. 2023 Mar 22;7:e38125. doi: 10.2196/38125 (PMC10131747; doi:10.2196/38125)
Supplement: Multimedia Appendix 1 [file formative_v7i1e38125_app1.docx]

## Appendix 1 Annotation guidelines

- Stated as being certain: When a finding is uncertain, it is not considered in staging
- Secondary tumor ipsilateral: size > 1,0 cm: A nodule should be larger than 1 cm to be considered in staging
- Atelectasis by tumor: Atelectasis can have different causes. Only tumor related atelectasis is considered in staging.
- Satellite nodules only when in the same lobe: The location of the satellite nodule should be in same lobe as the tumor, the term is inconsistently used.
- Enlarged lymph node: size >1,0 cm: We consider lymph nodes enlarged if its size is larger than 1 cm
