# Supplementary material for: How Natural Language Processing Can Aid With Pulmonary Oncology Tumor Node Metastasis Staging From Free-Text Radiology Reports: Algorithm Development and Validation
Source: JMIR Form Res. 2023 Mar 22;7:e38125. doi: 10.2196/38125 (PMC10131747; doi:10.2196/38125)
Supplement: Multimedia Appendix 2 [file formative_v7i1e38125_app2.docx]

## Appendix 2 Concept synonyms

*Regular expressions used for classification concepts tumor and involvement, corresponding SNOMED CT concepts*

General

| concept | regular expression | SNOMED CT concept |
| --- | --- | --- |
| tumor | (massa\|tumor\|nodu\|haard\|carcino\|laesie\|letsel\|\brip\b\|\bRIP\b\|ruimte(-)?innemend process\|maligniteit\|verdicht\|indicht) | 108369006 \| Neoplasm (morphologic abnormality) |
| involvement | aan(ge)?tast\|destructie\|(door\|in)(ge)?groei\|uitbreiding\|betrokken\|invade\|invasie\|induratie\|groeit (door\|in)\|betrekt | 248448006 \| Involved (qualifier value) |
| lymph nodes | lymf\|klier | 59441001 \| Structure of lymph node (body structure) |

T2 Presence

| concept | regular expression | SNOMED CT concept |
| --- | --- | --- |
| main_bronchus | (centrale\|hoofd\|hilair\|hilaire)/s?bronch | 102297006 \| Main bronchus structure (body structure) |
| visceral_pleura | pleura\|longvlies | 81623005 \| Visceral pleura structure (body structure) |

T2 Involvement

| concept | regular expression | SNOMED CT concept |
| --- | --- | --- |
| atelectasis | atelect\|samengevallen | 46621007 \| Atelectasis (disorder) |
| obstructive_pneumonitis | obstructieve pneumoni\|infecti(e\|ë)u(s\|z)e (verander\|component) | 205237003 \| Pneumonitis (disorder) |

T3 Involvement

| concept | regular expression | SNOMED CT concept |
| --- | --- | --- |
| chest_wall | borst.*wan\|thorax.*wan\|rib\|costa | 78904004 \| Chest wall structure (body structure) |
| nervus_phrenicus | nervus.*(phrenicus\|frenicus) | 50230006 \| Structure of phrenic nerve (body structure) |
| parietale_pericard | pericard | 76848001 \| Pericardial structure (body structure) |

T3 Presence

| concept | regular expression | SNOMED CT concept |
| --- | --- | --- |
| satellite_nodule | satelliet | 396408009 \| Specimen involvement by satellite nodule(s) present (finding) |

T4 Involvement

| concept | regular expression | SNOMED CT concept |
| --- | --- | --- |
| diaphragm | diafragm\|middenrif | 5798000 \| Diaphragm structure (body structure) |
| mediastinum | mediast | 72410000 \| Mediastinal structure (body structure) |
| heart | hart\|epicard | 80891009 \| Heart structure (body structure) |
| great vessels | grote vaten\|centrale vaten\|aorta(?!boog)\|vena cava\|vcs\|VCS | 3711007 \| Structure of great blood vessel (organ) (body structure) |
| trachea | trachea\|luchtpijp | 44567001 \| Tracheal structure (body structure) |
| recurrent_laryngeal_nerve | recurrent laryngeal nerve\|laryngeal nerve\| nervus laryngeus recurrens | 731050007 \| Entire recurrent laryngeal nerve (body structure) |
| oesophagus | slok\|oesof\|oesoph | 32849002 \| Esophageal structure (body structure) |
| vertebral body | wervel\|vertebra | 3572006 \| Structure of body of vertebra (body structure) |
| carina | carina | 28700002 \| Structure of carina of trachea (body structure) |

T4 Tumor in different lobes

| concept | regular expression | SNOMED CT concept |
| --- | --- | --- |
| superior_lobe_left | linker\s{0,1}bovenkwab\|boven\s{0,1}kwab links\|\bLBK\b\|\bLBL\b | 44714003 \| Structure of upper lobe of left lung (body structure) |
| superior_lobe_right | rechter\s{0,1}bovenkwab\|boven\s{0,1}kwab rechts\|\bRBK\b\|\bRBL\b | 362898004 \| Structure of upper lobe of right lung (body structure) |
| middle_lobe | midde.*(kwab\|lob)\|\bMK\b\|\bML\b | 72481006 \| Structure of middle lobe of right lung (body structure) |
| inferior_lobe_right | rechter\s{0,1}onder\s{0,1}(kwab\|lob)\|  onder\s{0,1}(kwab\|lob) recht\|\bROK\b\|\bROL\b | 266005 \| Structure of lower lobe of right lung (body structure) |
| inferior_lobe_left | linker\s{0,1}onder\s{0,1}(kwab\|lob)\|  onder\s{0,1}(kwab\|lob) link\|\bLOK\b\|\bLOL\b | 41224006 \| Structure of lower lobe of left lung (body structure) |

**N additions**

Lateralization

| concept | regular expression | SNOMED CT concept |
| --- | --- | --- |
| left | links\|linker\|\bL\b\|(?<=\d\s)(l\|L)\b\|(?<=\d)(l\|L)\b | 31156008 \| Structure of left half of body (body structure) |
| right | rechts\|rechter\|\bR\b\|(?<=\d\s)(r\|R)\b\|(?<=\d)(r\|R)\b | 85421007 \| Structure of right half of body (body structure) |
| ipsilateral | ipsilater | 255208005 \| Ipsilateral (qualifier value) |
| contralateral | contralater | 255209002 \| Contralateral (qualifier value) |
| bilateral | bilater\|beide.*zijd\|weerskanten\|alle.*(?<=station)\|(?<=level)\|(?<=regio)\|(?<=zone)\|(links\|linker\|linkerzijde\|linkerkant)\s(en\|als)\s(rechts\|rechter\|rechterzijde\|rechterkant)\|(rechts\|rechter\|rechterzijde\|rechterkant)\s(en\|als)\s(links\|linker\|linkerzijde\|linkerkant)' | 51440002 \| Right and left (qualifier value) |

Pathological lymph nodes

| concept | regular expression | SNOMED CT concept |
| --- | --- | --- |
| lymph_nodes_pathologic_adj | pathologisch\|vergroot\|vergrot\|afwijkend\|metastase | 29458008 \| Pathologic (qualifier value) |
| lymph_adenopathy | lymfadenopathi\|adenopathi\|klier\s?metastase | 30746006 \| Lymphadenopathy (disorder) |

Levels

| concept | regular expression | SNOMED CT concept |
| --- | --- | --- |
| thorax-station-1 | ((?<=station)\|(?<=level)\|(?<=regio)\|(?<=zone))s?\s?\b1\|(supra\|retro)-{0,1}\s?claviculair\|cervicaal\|sternaal\|\b(1\|I\b\|Ⅰ\b)\s?(?=(r\b\|R\b\|rechts\|l\b\|L\b\|links)) | 127926002 \| Highest mediastinal lymph node (body structure) |
| thorax-station-2 | ((?<=station)\|(?<=level)\|(?<=regio)\|(?<=zone))s?\s?\b2\|para-{0,1}\s?tracheaal\|hoog paratracheaal\|\b(2\|II\b\|Ⅱ)\s?(?=(r\b\|R\b\|rechts\|l\b\|L\b\|links)) | 127927006 \| Upper paratracheal lymph node (mediastinal) (body structure) |
| thorax-station-3a | ((?<=station)\|(?<=level)\|(?<=regio)\|(?<=zone))s?\s?(3\|III\b\|Ⅲ)a\|pre-{0,1}\s?vasculair | 263846009 \| Prevascular (qualifier value) 🡪 no lymph node  *127930004 \| Prevascular/retrotracheal lymph node (body structure) = AJCC 3*  *Single concept different classification* |
| thorax-station-3p | ((?<=station)\|(?<=level)\|(?<=regio)\|(?<=zone))s?\s?(3\|III\b\|Ⅲ)p\|pre-{0,1}\s?vertrebra | 196446004 \| Structure of prevertebral lymph node (body structure)  *127930004 \| Prevascular/retrotracheal lymph node (body structure) = AJCC 3*  *Single concept different classification* |
| thorax-station-4 | ((?<=station)\|(?<=level)\|(?<=regio)\|(?<=zone))s?\s?\b(4\|IV\b\|Ⅳ)\|para-{0,1}\s?trachea\|mediastinale (?=(lymf\|klier\|adenopat\|patholo\|vergro))\|mediastinaal (?=(lymf\|klier\|adenopat\|patholo\|vergro))\|((?<=klier)\|(?<=klieren)) mediastinaal\|\b4\s?(?=(r\b\|R\b\|rechts\|l\b\|L\b\|links)) | 127933002 \| Lower paratracheal lymph node, superior group (body structure) |
| thorax-station-5 | ((?<=station)\|(?<=level)\|(?<=regio)\|(?<=zone))s?\s?\b(5\|V\b\|Ⅴ)\|sub-{0,1}\s?aortaal\|(AP\|aorta-{0,1}\s?pulmona)-{0,1}\s?(window\|venster) | 127938006 \| Lymph node of aortopulmonary window (body structure) |
| thorax-station-6 | ((?<=station)\|(?<=level)\|(?<=regio)\|(?<=zone))s?\s?\b(6\|VI\b\|Ⅵ)\|para-{0,1}\s?aorta | 127939003 \| Structure of para-aortic lymph node of anterior mediastinum (body structure) |
| thorax-station-7 | ((?<=station)\|(?<=level)\|(?<=regio)\|(?<=zone))s?\s?\b(7\|VII\b\|Ⅶ)\|sub-{0,1}\s?carina\|pre-{0,1}\s?carina\|7\s?(?=(r\b\|R\b\|rechts\|l\b\|L\b\|links)) | 28330007 \| Structure of subcarinal lymph node (body structure) |
| thorax-station-8 | ((?<=station)\|(?<=level)\|(?<=regio)\|(?<=zone))s?\s?\b(8\|VIII\|ⅤⅠⅠⅠ\|Ⅷ)\|para-{0,1}\s?(oesof\|oesoph)\|8\s?(?=(r\b\|R\b\|rechts\|l\b\|L\b\|links)) | 127940001 \| Paraesophageal lymph node below carina (body structure) |
| thorax-station-9 | ((?<=station)\|(?<=level)\|(?<=regio)\|(?<=zone))s?\s?\b(9\|IX\b\|Ⅸ)\|pulmonair ligament\|9\s?(?=(r\b\|R\b\|rechts\|l\b\|L\b\|links)) | 127941002 \| Lymph node of the pulmonary ligament (body structure) \| |
| thorax-station-10 | ((?<=station)\|(?<=level)\|(?<=regio)\|(?<=zone))s?\s?\b10\|hilair\|hilus\|(10\|X\b\|Ⅹ)\s?(?=(r\b\|R\b\|rechts\|l\b\|L\b\|links)) | 53074004 \| Structure of hilar lymph node (body structure) |
